# Supplementary material for: Maltodextrin transport in the extremely thermophilic, lignocellulose degrading bacterium Anaerocellum bescii (f. Caldicellulosiruptor bescii)
Source: J Bacteriol. 2025 Apr 30;207(5):e00401-24. doi: 10.1128/jb.00401-24 (PMC12096829; doi:10.1128/jb.00401-24)
Supplement: Supplemental figures and tables — Figures S1 to S6; Tables S1 to S3. [file jb.00401-24-s0001.pdf]

Supplementary Information for:

Maltodextrin Transport in the Extremely Thermophilic, Lignocellulose Degrading Bacterium *Anaerocellum bescii* (f. *Caldicellulosiruptor bescii*)

Hansen Tjo [1], Virginia Jiang [1], Jerelle A. Joseph [1,2], Jonathan M. Conway [1,2,3,4,5,#]

Author Affiliations:
[1] Department of Chemical and Biological Engineering, Princeton University, Princeton, NJ 08544, USA
[2] Omenn-Darling Bioengineering Institute, Princeton University, Princeton, NJ 08544, USA
[3] Department of Molecular Biology, Princeton University, Princeton NJ 08544, USA
[4] Andlinger Center for Energy and the Environment, Princeton University, Princeton, NJ 08544, USA
[5] High Meadows Environmental Institute, Princeton University, Princeton, NJ 08544, USA

[#]Corresponding Author: Jonathan M. Conway jmconway@princeton.edu

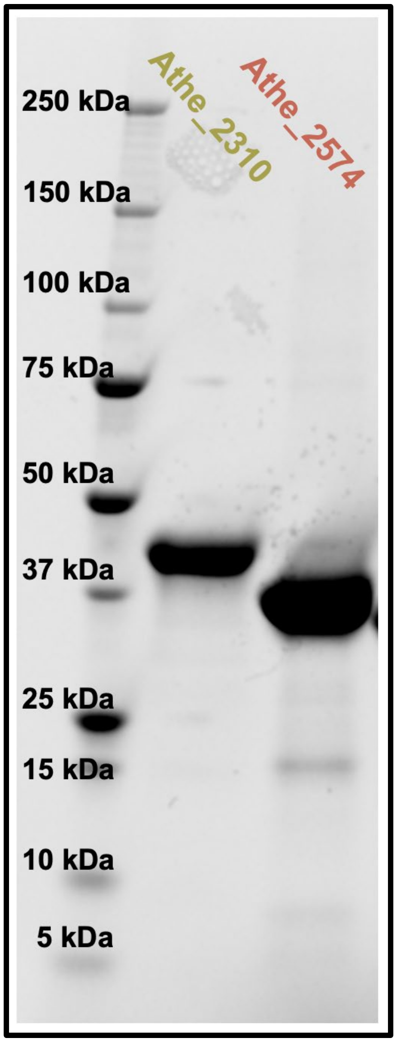

Figure S1: SDS-PAGE gel of purified Athe_2310 and Athe_2574 used in this study.

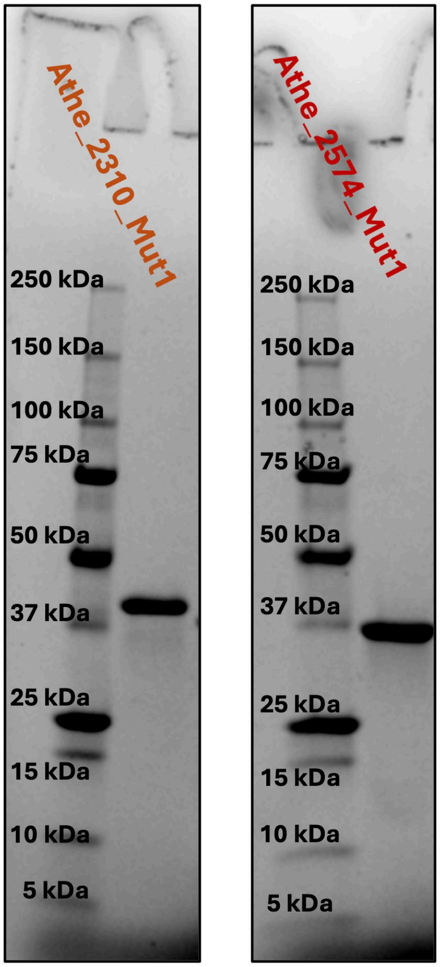

Figure S2: SDS-PAGE gel of purified MBP mutants Athe_2310_Mut1 and Athe_2574_Mut1 used in this study.

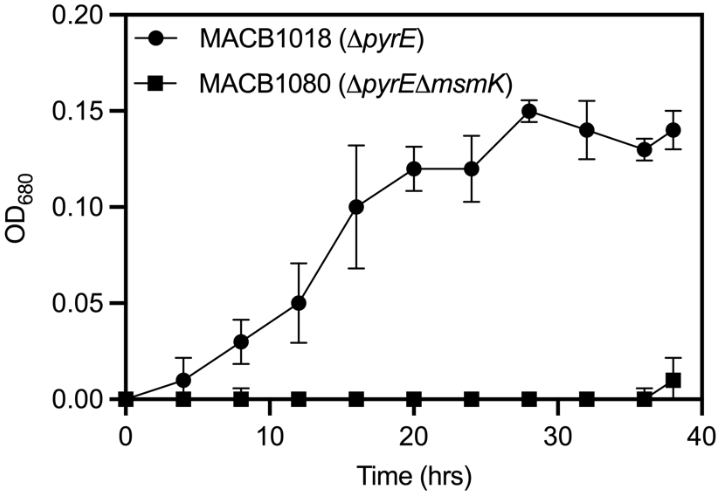

Figure S3: Growth curves of *msmK* deletion *A. bescii* strain MACB1080 (Δ*pyrE*Δ*msmK*) and parent strain MACB1018 (Δ*pyrE*) on maltose substrate. MACB1080 is unable to grow on maltose, indicating that the Athe_2308 – 2310 and Athe_2574 – 2578 transporters rely on MsmK for ATP-powered maltodextrin uptake. All growth curves were performed in triplicate; error bars indicate the standard deviation about the mean.

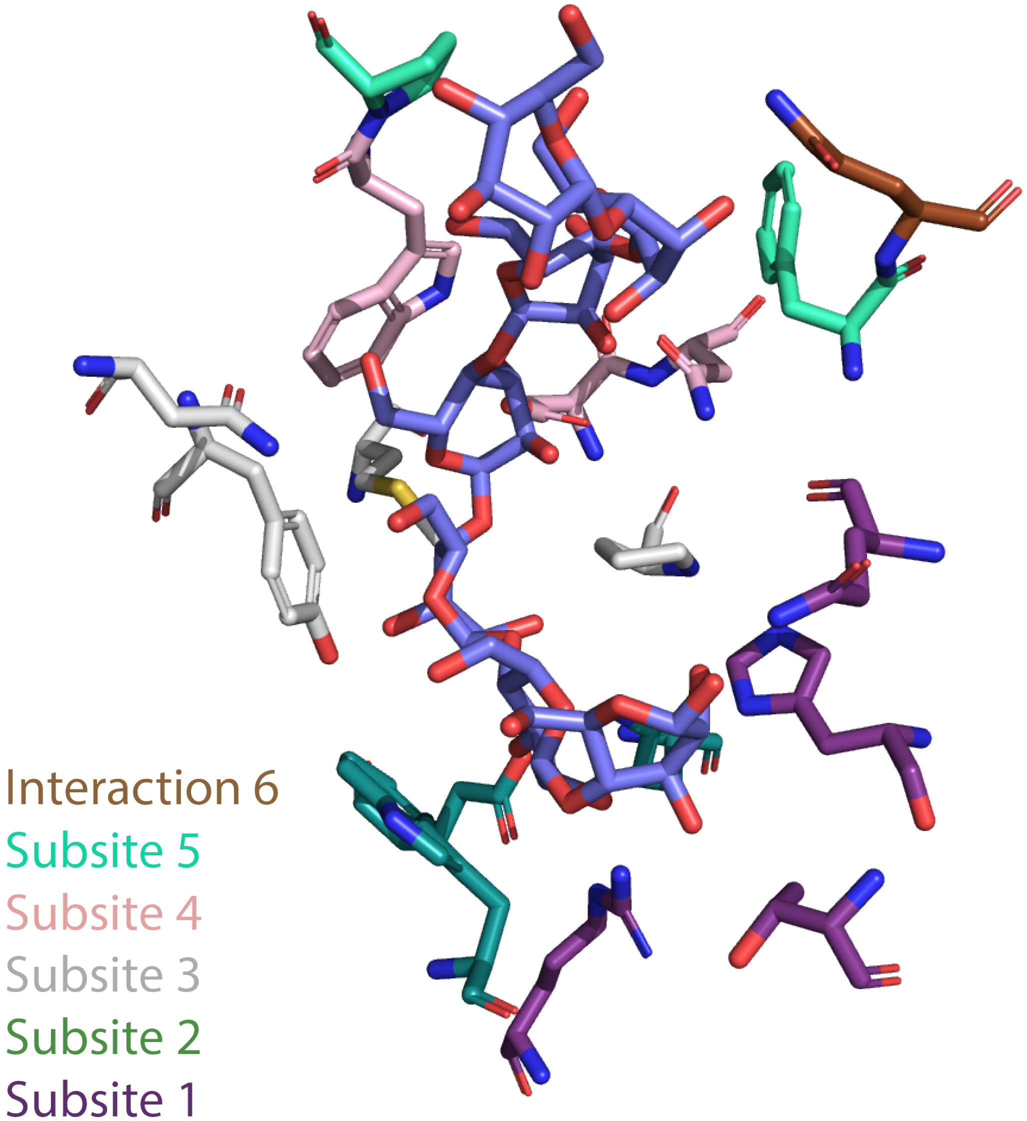

Figure S4: Predicted orientation of maltoheptaose (G7) binding in an AlphaFold2 predicted structure of Athe_2574. Additional glucosyl residues that cannot occupy one of the existing five subsites within the binding pocket are stabilized by hydrogen bonding interactions with residues on the protein surface, such as shown by Interaction 6.

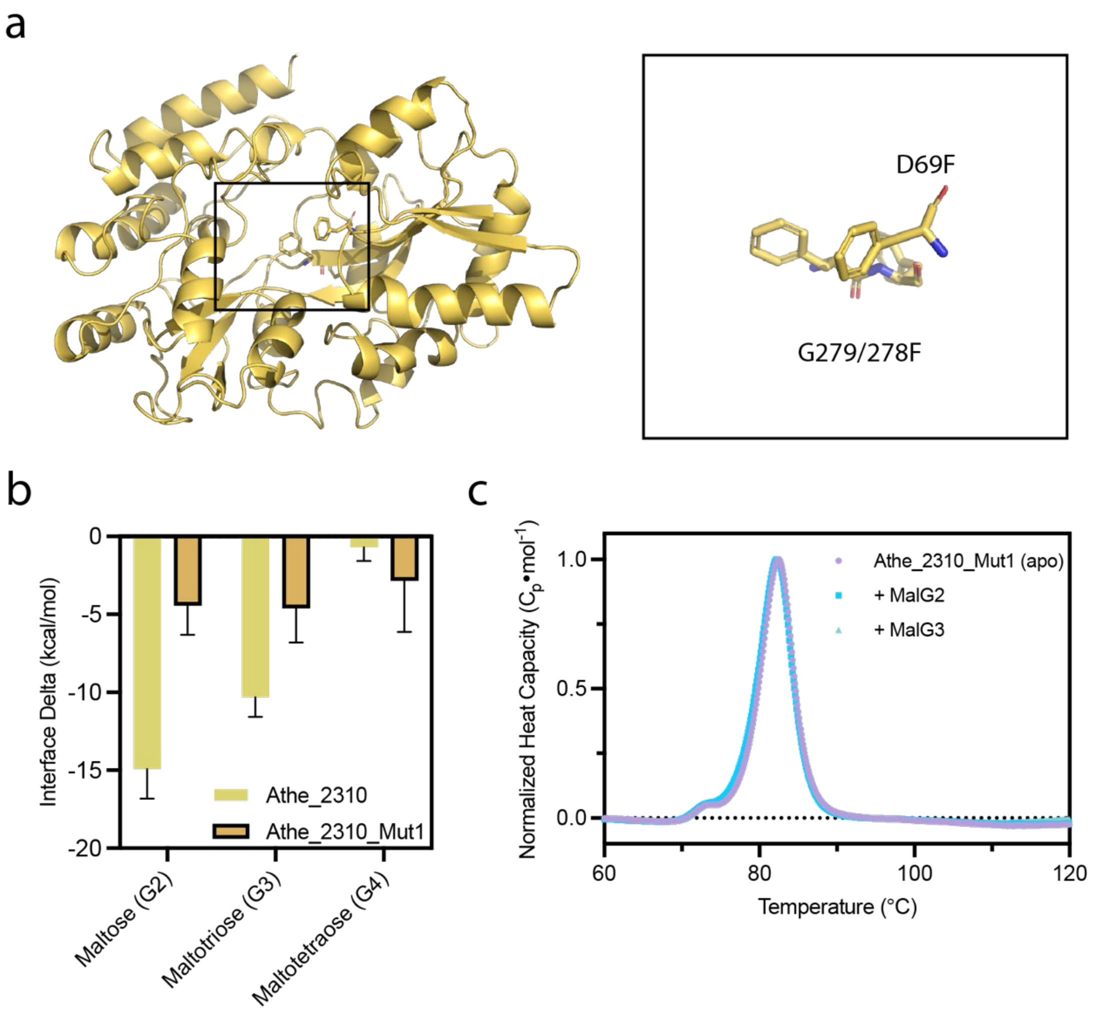

Figure S5: a) AlphaFold2 predicted structure of Athe_2310_Mut1. A close-up view of the binding pocket illustrates all mutated residues to perturb ligand coordination. b) Interface energy deltas, corresponding to free energies of binding, show that simulated Athe_2310_Mut1 binds maltodextrin substrates (G2 – G4) worse than wild-type Athe_2310 ($n = 200$). c) Normalized DSC screens of Athe_2310_Mut1 mixed with its cognate maltodextrins show minimal changes to its melting temperature, indicating an absence of ligand-induced conformational change as shown in Figure 4.

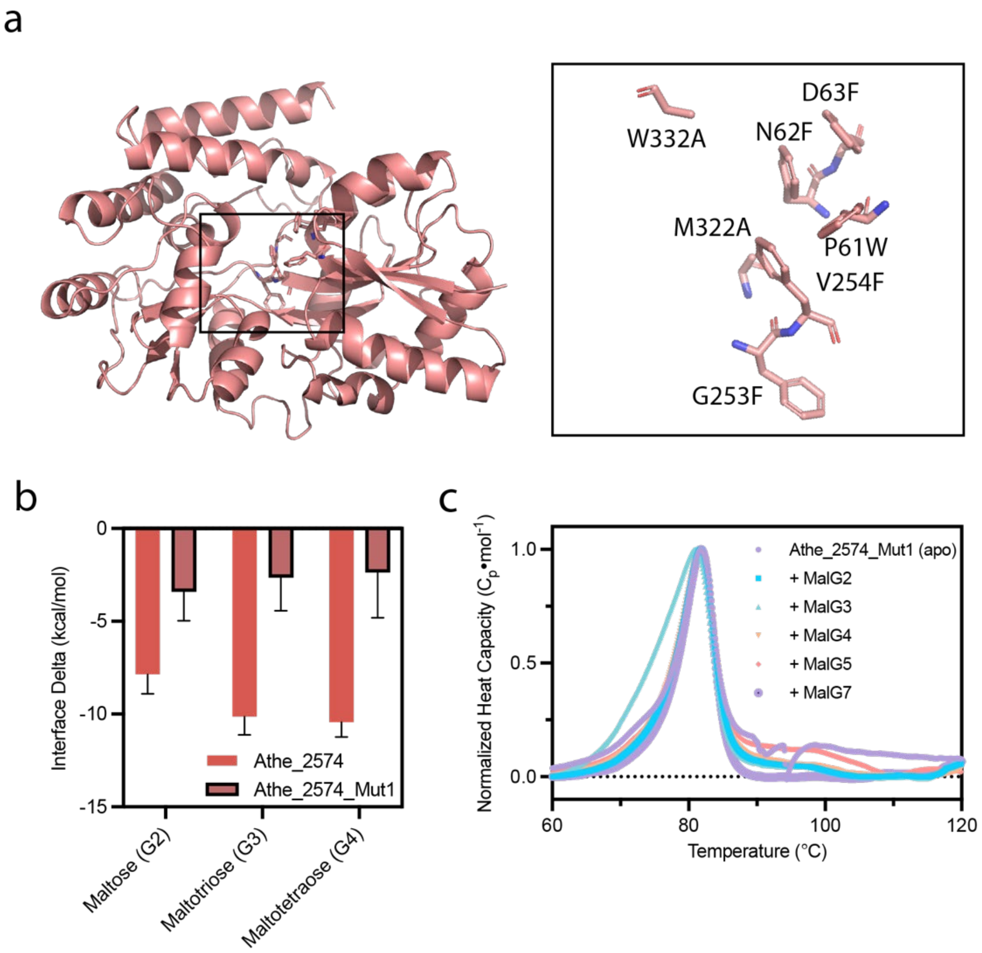

Figure S6: a) AlphaFold2 predicted structure of Athe_2574_Mut1. A close-up view of the binding pocket illustrates all mutated residues to perturb ligand coordination. b) Interface energy deltas, corresponding to free energies of binding, show that simulated Athe_2574_Mut1 binds its maltodextrin substrates (G2 – G4) worse than wild-type Athe_2574 ($n = 200$). c) Normalized DSC screens of Athe_2574_Mut1 mixed with various maltodextrins show minimal changes to its melting temperature, indicating an absence of ligand-induced conformational change as shown in Figure 4.

Table S1: Table of primers used in this study.

Primer	DNA Sequence (5' – 3')	Application
HT001_F	GCTGCCACCGCTGAGCAATAACTAG	pRSF1-b Vector.FOR (backbone linearization)
HT002_F	cttgtcgtcgtcatccacgtgatg	pRSF1-b Vector.REV (backbone linearization)
HT005	CACGTGGATGACGACGACAAGGCTTCTAAAAAACAGGTCACAATTACTTATGTTCG	pHT002 Fragment FOR (Insert amplification from *A. bescii* genomic DNA)
HT006	TTGCTCAGCGGTGGCAGCTTACTTTGAAGTTTTTACAACTTCTTTTAATTTCTTGTCC	pHT002 Fragment REV (Insert amplification from *A. bescii* genomic DNA)
HT134	tgagatccggctgctaacaaag	pCri8a Vector.FOR (backbone linearization)
HT135	catggcgccctggaagtaaag	pCri8a Vector.REV (backbone linearization)
HT136	tttacttccagggcgccatggttACATCCAAAAAACAGCTTGTTGTC	pHT003a Fragment.FOR Athe_2574 (Insert amplification from *A. bescii* genomic DNA)
HT137	ctttgttagcagccggatctcaTTATTGCATCTGAGCAATACCTTGCTTG	pHT003a Fragment.REV Athe_2574 (Insert amplification from *A. bescii* genomic DNA)

Table S2: Amino acid sequences of all proteins analyzed in this study. Residues comprising the signal peptide sequence are marked in red.

Protein	Amino Acid Sequence
Athe_2310<br>(native with signal peptide)<br>(JGI Accession:<br>YP_002574153)	MKRFIAVMVLIAFSVGLFLAFGPANSNAASKKQVTITYVRGKDETHATE<br>KIIKEFMKKNPDINVIYKENPSDTGQNHDQLVTVLSAGGSDIDVFDMDV<br>IWPAEFAQAGYTLPLDRFIKRDKTNLNDYIKGTIDAARFKGQMWAFPR<br>FIDAGLLYYRKDIVPQNELPKTWDDLIKVAKKYKGKNGTKYGFLMQAK<br>QYEGLVCDAIEYIASYGGKVVDESGNIVVNNQGTIDGLNMMRKVITSGI<br>VPPNINTFTEVETHTAFINGLSVFARNWPYMWAMINSPQSKVRGKVGI<br>LPLPKGSKGSAACLGGWMVGINKFSKNPEASWRLLKFLVQKEGQKL<br>MAIYNGNVPVYKPLFNDKDVIKANPLIGDKKFIEAILAAVPRPVSPIYPKI<br>SDVMQIELSNIVNGKKDVKTAVADMDKKLKEVVKTSK
Athe_2574<br>(native with signal peptide)<br>(JGI Accession:<br>YP_002574412)	MKNLKRILTVALIITFAVVALIPLSGVFATSKKQLVVWSHLTQDEVKALQ<br>PIADKWGKENGYTVKVITDQGSFQSFQTAAMSGKGPDIMFGIPHDNL<br>GAFWKAKLLEAVPANLIDKKNFVSTALDACSFEGKLYALPIAMETYALF<br>YNTSKVKEAPKTMSQLITLAKKYGFMYDVNNFYFSFAFIAQNGGYVFK<br>NKGGSLDPNDIGLATNGAIKGLSLIRDFVQTYKFMPKDIKGDIAKGNFQ<br>NQKIAFYISGPWDVQDFIKAKVPFAVAPLPKTDDGKPTPSFVGVQAAF<br>VSAKSKNKDAAFKLMKYLVENSALTLFKVGHRIPVLNKVLTSSEVKAD<br>KIMSAFAEQAKVGIPMPNIPEMSAVWPVANNALSLITTGKATPKQAAD<br>AMVKQIKQGIAQMQ
Athe_2310<br>(cloned in *E. coli*)	MAHHHHHHVDDDDKASKKQVTITYVRGKDETHATEKIIKEFMKKNPDI<br>NVIYKENPSDTGQNHDQLVTVLSAGGSDIDVFDMDVIWPAEFAQAGY<br>TLPLDRFIKRDKTNLNDYIKGTIDAARFKGQMWAFPRFIDAGLLYYRKD<br>IVPQNELPKTWDDLIKVAKKYKGKNGTKYGFLMQAKQYEGLVCDAIEY<br>IASYGGKVVDESGNIVVNNQGTIDGLNMMRKVITSGIVPPNINTFTEVE<br>THTAFINGLSVFARNWPYMWAMINSPQSKVRGKVGILPLPKGSKGSA<br>ACLGGWMVGINKFSKNPEASWRLLKFLVQKEGQKLMAIYNGNVPVY<br>KPLFNDKDVIKANPLIGDKKFIEAILAAVPRPVSPIYPKISDVMQIELSNIV<br>NGKKDVKTAVADMDKKLKEVVKTSK
Athe_2574<br>(cloned in *E. coli*)	MGSSHHHHHHSSGENLYFQGAMVTSKKQLVVWSHLTQDEVKALQPI<br>ADKWGKENGYTVKVITDQGSFQSFQTAAMSGKGPDIMFGIPHDNLGA<br>FWKAKLLEAVPANLIDKKNFVSTALDACSFEGKLYALPIAMETYALFYN<br>TSKVKEAPKTMSQLITLAKKYGFMYDVNNFYFSFAFIAQNGGYVFKNK<br>GGSLDPNDIGLATNGAIKGLSLIRDFVQTYKFMPKDIKGDIAKGNFQNQ<br>KIAFYISGPWDVQDFIKAKVPFAVAPLPKTDDGKPTPSFVGVQAAFVS<br>AKSKNKDAAFKLMKYLVENSALTLFKVGHRIPVLNKVLTSSEVKADKIM<br>SAFAEQAKVGIPMPNIPEMSAVWPVANNALSLITTGKATPKQAADAMV<br>KQIKQGIAQMQ
Athe_2310_<br>pointmut #D69F G279F<br>G278F (Athe_2310_Mut1)	MAHHHHHHVDDDDKASKKQVTITYVRGKDETHATEKIIKEFMKKNPDI<br>NVIYKENPSDTGQNHDQLVTVLSAGGSDIDVFDMFVIWPAEFAQAGY<br>TLPLDRFIKRDKTNLNDYIKGTIDAARFKGQMWAFPRFIDAGLLYYRKD<br>IVPQNELPKTWDDLIKVAKKYKGKNGTKYGFLMQAKQYEGLVCDAIEY<br>IASYGGKVVDESGNIVVNNQGTIDGLNMMRKVITSGIVPPNINTFTEVE<br>THTAFINGLSVFARNWPYMWAMINSPQSKVRGKVGILPLPKGSKGSA<br>ACLFFWMVGINKFSKNPEASWRLLKFLVQKEGQKLMAIYNGNVPVYK

	PLFNDKDVIKANPLIGDKKFIEAILAAVPRPVSPIYPKISDVMQIELSNIV<br>NGKKDVKTAVADMDKKLKEVVKTSK
Athe_2574_pointmut<br>#G253F V254F P61W N63F<br>D64F M322A W332A<br>(Athe_2574_Mut1)	MGSSHHHHHHSSGENLYFQGAMKQLVVWSHLTQDEVKALQPIADK<br>WGKENGYTVKVITDQGSFQSFQTAAMSGKGPDIMFGIFHFFLGAFWK<br>AKLLEAVPANLIDKKNFVSTALDACSFEGKLYALPIAMETYALFYNTSK<br>VKEAPKTMSQLITLAKKYGFMYDVNNFYFSFAFIAQNGGYVFKNKGG<br>SLDPNDIGLATNGAIKGLSLIRDFVQTYKFMPKDIKGDIAKGNFQNQKI<br>AFYISGPWDVQDFIKAKVPFAVAPLPKTDDGKPTPSFFFVQAAFVSAK<br>SKNKDAAFKLMKYLVENSALTLFKVGHRIPVLNKVLTSSEVKADKIMSA<br>FAEQAKVGIPAPNIPEMSAVAPVANNALSLITTGKATPKQAADAMVKQI<br>KQGIAQM
TmMBP3 (PDB: 6DTQ)	MAVKITMTSGGVGKELEVLKKQLEMFHQQYPDIEVEIIPMPDSSTERH<br>DLYVTYFAAGETDPDVLMLDVIWPAEFAPFLEDLTADKDYFELGEFLP<br>GTVMSVTVNGRIVAVPWFTDAGLLYYRKDLLEKYGYDHAPRTWDELV<br>EMAKKISQAEGIHGFVWQGARYEGLVCDFLEYLWSFGGDVLDESGK<br>VVIDSPEAVAALQFMVDLIYKHKVTPEGVTTYMEEDARRIFQNGEAVF<br>MRNWPYAWSLVNSDESPIKGKVGVAPLPMGPGGRRAATLGGWVLGI<br>NKFSSPEEKEAAKKLIKFLTSYDQQLYKAINAGQNPTRKAVYKDPKLK<br>EAAPFMVELLGVFINALPRPRVANYTEVSDVIQRYVHAALTRQTTSED<br>AIKNIAKELKFLLGQHHHHHH
TMBP (PDB: 1EU8)	IEEGKIVFAVGGAPNEIEYWKGVIAEFEKKYPGVTVELKRQATDTEQR<br>RLDLVNALRGKSSDPDVFLMDVAWLGQFIASGWLEPLDDYVQKDNY<br>DLSVFFQSVINLADKQGGKLYALPVYIDAGLLYYRKDLLEKYGYSKPP<br>ETWQELVEMAQKIQSGERETNPNFWGFVWQGKQYEGLVCDFVEYV<br>YSNGGSLGEFKDGKWVPTLNKPENVEALQFMVDLIHKYKISPPNTYT<br>EMTEEPVRLMFQQGNAAFERNWPYAWGLHNADDSPVKGKVGVAPL<br>PHFPGHKSAATLGGWHIGISKYSDNKALAWEFVKFVESYSVQKGFAM<br>NLGWNPGRVDVYDDPAVVSKSPHLKELRAVFENAVPRPIVPYYPQLS<br>EIIQKYVNSALAGKISPQEALDKAQKEAEELVKQYS
TtMBP (PDB: 6J9W)	QSGPVIRVAGDSTAVGEGGRWMKEMVEAWGKKTGTRVEYIDSPADT<br>NDRLALYQQYWAARSPDVDVYMIDVIWPGIVAPHALDLKPYLTEAELK<br>EFFPRIVQNNTIRGKLTSLPFFTDAGILYYRKDLLEKYGYTSPPRTWNE<br>LEQMAERVMEGERRAGNRDFWGFVFQGKPYEGLTCDALEWIYSHG<br>GGRIVEPDGTISVNNGRAALALNRAHGWVGRIAPQGVTSYAEEEARN<br>VWQQGNSLFMRNWPYAYALGQAEGSPIRGKFGVTVLPKASADAPNA<br>ATLGGWQLMVSAYSRYPKEAVDLVKYLASYEVQKDNAVRLSRLPTRP<br>ALYTDRDVLARNPWFRDLLPVFQNAVSRPSDVAGARYNQVSEAIWTE<br>VHSVLTGRKKGEQAVRDLEARIRRILRHHHHHH
PfuMBP (PDB: 1ELJ)	MKIEEGKVVIWHAMQPNELEVFQSLAEEYMALCPEVEIVFEQKPNLED<br>ALKAAIPTGQGPDLFIWAHDWIGKFAEAGLLEPIDEYVTEDLLNEFAPM<br>AQDAMQYKGHYYALPFAAETVAIIYNKEMVSEPPKTFDEMKAIMEKYY<br>DPANEKYGIAWPINAYFISAIAQAFGGYYFDDKTEQPGLDKPETIEGFK<br>FFFTEIWPYMAPTGDYNTQQSIFLEGRAPMMVNGPWSINDVKKAGIN<br>FGVVPLPPIIKDGKEYWPRPYGGVKLIYFAAGIKNKDAAWKFAKWLTT<br>SEESIKTLALELGYIPVLTKVLDDPEIKNDPVIYGFGQAVQHAYLMPKS<br>PKMSAVWGGVDGAINEILQDPQNADIEGILKKYQQEILNNMQG

TmMBP1 (PDB: 6DTU)	MQPKLTIWCSEKQVDILQKLGEEFKAKYGVEVEVQYVNFQDIKSKFLT<br>AAPEGQGADIIVGAHDWVGELAVNGLIEPIPNFSDLKNFYETALNAFSY<br>GGKLYGIPYAMEAIALIYNKDYVPEPPKTMDELIEIAKQIDEEFGGEVR<br>GFITSAAEFYYIAPFIFGYGGYVFKQTEKGLDVNDIGLANEGAIKGVKLL<br>KRLVDEGILDPSDNYQIMDSMFREGQAAMIINGPWAIKAYKDAGIDYG<br>VAPIPDLEPGVPARPFVGVQGFMVNAKSPNKLLAIEFLTSFIAKKETMY<br>RIYLGDPRLPSRKDVLELVKDNPDVVGFTLSAANGIPMPNVPQMAAV<br>WAAMNDALNLVVNGKATVEEALKNAVERIKAQIQS
TmMBP2 (PDB: 6DTS)	MQTKLTIWCSEKQVDILQKLGEEFKAKYGIPVEVQYVDFGSIKSKFLTA<br>APQGQGADIIVGAHDWVGELAVNGLIEPIPNFSDLKNFYDTALKAFSY<br>GGKLYGVPYAMEAVALIYNKDYVDSVPKTMDELIEKAKQIDEEYGGEV<br>RGFIYDVANFYFSAPFILGYGGYVFKETPQGLDVTDIGLANEGAVKGA<br>KLIKRMIDEGVLTPGDNYGTMDSMFKEGLAAMIINGLWAIKSYKDAGIN<br>YGVAPIPELEPGVPAKPFVGVQGFMINAKSPNKVIAMEFLTNFIARKET<br>MYKIYLADPRLPARKDVLELVKDNPDVVAFTQSASMGTPMPNVPEMA<br>PVWSAMGDALSIIINGQASVEDALKEAVEKIKAQIEKGSHHHHHH

Table S3: DNA sequences of gene synthesis for maltodextrin-binding protein mutants.

Protein	DNA Sequence
Athe_2310_<br>pointmut #D69F G279F<br>G278F (Athe_2310_Mut1)	GCGAGCAAAAAACAGGTAACCATTACCTATGTGCGTGGCAAAGAT<br>GAAACGCATGCGACCGAAAAAATTATTAAAGAGTTTATGAAAAAA<br>ACCCGGATATTAACGTGATTTACAAAGAAAACCCGAGCGATACGG<br>GCCAGAACCATGATCAACTGGTTACCGTGCTGAGCGCAGGTGGCA<br>GCGATATTGATGTGTTCGATATGTTCGTGATCTGGCCGGCGGAATT<br>CGCGCAGGCGGGCTATACCCTGCCGCTGGATCGCTTCATTAAACG<br>CGATAAAACCAATCTGAACGATTACATTAAAGGCACCATTGATGCG<br>GCCCGCTTTAAAGGCCAGATGTGGGCGTTTCCGCGCTTCATTGAT<br>GCGGGCCTGCTGTATTATCGTAAAGATATTGTGCCGCAGAACGAA<br>CTGCCGAAAACCTGGGACGATCTGATTAAAGTGGCCAAAAAATATA<br>AAGGCAAAAATGGCACCAAATACGGCTTTCTGATGCAGGCGAAAC<br>AGTATGAAGGCCTGGTGTGCGATGCGATTGAATACATTGCGTCTTA<br>TGGCGGCAAAGTGGTGGATGAAAGCGGAAATATTGTGGTGAATAA<br>TCAGGGCACCATTGATGGCTTAAATATGATGCGCAAAGTGATTACC<br>AGCGGCATTGTGCCGCCGAACATTAACACGTTCACCGAAGTGGAA<br>ACGCATACCGCGTTTATTAATGGCCTGAGCGTATTTGCGCGCAACT<br>GGCCGTATATGTGGGCGATGATTAACAGCCCGCAGAGCAAAGTGC<br>GCGGCAAAGTAGGCATTCTGCCGCTGCCGAAAGGCAGCAAAGGC<br>AGCGCCGCGTGTCTGTTTTTCTGGATGGTTGGCATTAACAAATTTA<br>GCAAAAACCCGGAAGCGTCATGGCGCCTGCTGAAATTTCTGGTGC<br>AGAAAGAAGGCCAAAAACTGATGGCGATCTATAACGGTAACGTGC<br>CGGTGTATAAACCGCTGTTTAACGATAAAGATGTTATTAAAGCGAA<br>CCCGCTGATCGGCGATAAAAAATTCATTGAAGCGATTCTGGCGGC<br>AGTGCCGCGCCCGGTGAGCCCGATCTATCCGAAAATTAGCGATGT<br>GATGCAGATCGAACTGAGCAACATTGTAAATGGCAAAAAAGATGTG<br>AAAACCGCAGTGGCGGATATGGATAAAAAACTGAAAGAAGTGGTG<br>AAAACCAGCAAATAA
Athe_2574_pointmut<br>#G253F V254F P61W N63F<br>D64F M322A W332A<br>(Athe_2574_Mut1)	AAACAGCTGGTGGTGTGGAGCCATCTGACCCAGGATGAAGTGAAA<br>GCGCTGCAGCCGATTGCGGATAAATGGGGTAAAGAAAACGGTTAT<br>ACCGTGAAAGTGATTACCGATCAGGGCAGCTTTCAGAGCTTCCAG<br>ACCGCCGCGATGAGTGGCAAAGGCCCGGATATTATGTTTGGCATT<br>TTTCATTTTTTTCTGGGCGCGTTCTGGAAAGCGAAACTGCTGGAAG<br>CGGTGCCGGCGAACCTGATTGATAAAAAAAACTTTGTTAGCACCG<br>CGCTGGATGCGTGTAGCTTTGAGGGCAAACTGTATGCGCTGCCGA<br>TCGCGATGGAAACCTACGCGCTGTTTTACAACACCAGCAAAGTGA<br>AAGAAGCGCCTAAAACCATGAGCCAGCTGATTACCCTGGCCAAAA<br>AATATGGCTTTATGTATGATGTGAACAATTTTTATTTTAGCTTTGCG<br>TTTATTGCGCAGAATGGCGGCTATGTGTTCAAAAACAAAGGCGGTT<br>CCCTGGATCCGAACGATATTGGCCTGGCGACCAACGGCGCCATTA<br>AAGGTCTGAGCCTGATTCGCGATTTTGTGCAGACCTATAAATTTAT<br>GCCGAAAGATATTAAAGGTGATATTGCCAAAGGCAATTTTCAGAAC<br>CAGAAAATTGCGTTCTATATTAGCGGCCCGTGGGATGTGCAGGAC<br>TTTATCAAAGCGAAAGTGCCGTTCGCGGTGGCCCCGCTGCCGAAA<br>ACCGATGATGGCAAACCGACCCCGAGCTTTTTTTTTGTGCAGGCG<br>GCGTTTGTGAGCGCGAAAAGCAAAAATAAAGATGCCGCGTTTAAA

	CTGATGAAATACCTGGTGGAAAACAGCGCCCTGACCCTGTTCAAA<br>GTTGGCCATCGTATTCCGGTCCTGAATAAAGTCCTGACCTCAAGC<br>GAAGTGAAAGCCGATAAAATTATGAGCGCGTTTGCCGAACAGGCG<br>AAAGTTGGGATCCCGGCGCCGAATATTCCGGAAATGTCGGCCGTG<br>GCCCCGGTGGCCAACAACGCGCTGAGCCTGATTACCACCGGCAA<br>AGCAACCCCGAAACAAGCAGCCGATGCGATGGTTAAACAGATTAA<br>ACAGGGCATCGCGCAGATGTAA
